# Genomic imprinting and genetic effects on muscle traits in mice

**DOI:** 10.1186/1471-2164-13-408

**Published:** 2012-08-20

**Authors:** Stefan Kärst, Ali R Vahdati, Gudrun A Brockmann, Reinmar Hager

**Affiliations:** 1 Department for Crop and Animal Sciences, Humboldt-University Berlin, Berlin, Germany; 2 Computational and Evolutionary Biology, Faculty of Life Sciences, University of Manchester, Manchester, M13 9PT, UK

## Abstract

**Background:**

Genomic imprinting refers to parent-of-origin dependent gene expression caused by differential DNA methylation of the paternally and maternally derived alleles. Imprinting is increasingly recognized as an important source of variation in complex traits, however, its role in explaining variation in muscle and physiological traits, especially those of commercial value, is largely unknown compared with genetic effects.

**Results:**

We investigated both genetic and genomic imprinting effects on key muscle traits in mice from the Berlin Muscle Mouse population, a key model system to study muscle traits. Using a genome scan, we first identified loci with either imprinting or genetic effects on phenotypic variation. Next, we established the proportion of phenotypic variation explained by additive, dominance and imprinted QTL and characterized the patterns of effects. In total, we identified nine QTL, two of which show large imprinting effects on glycogen content and potential, and body weight. Surprisingly, all imprinting patterns were of the bipolar type, in which the two heterozygotes are different from each other but the homozygotes are not. Most QTL had pleiotropic effects and explained up to 40% of phenotypic variance, with individual imprinted loci accounting for 4-5% of variation alone.

**Conclusion:**

Surprisingly, variation in glycogen content and potential was only modulated by imprinting effects. Further, in contrast to general assumptions, our results show that genomic imprinting can impact physiological traits measured at adult stages and that the expression does not have to follow the patterns of paternal or maternal expression commonly ascribed to imprinting effects.

## Background

Genomic imprinting is increasingly seen as an important epigenetic source of phenotypic variation that may act in addition to or in conjunction with genetic sources of variation [1-3]. Imprinting occurs when identical alleles are expressed depending on whether they were inherited from the father or the mother, thus causing a parent-of-origin-specific gene expression pattern where either the paternal allele is expressed and the maternal allele is silenced (paternal expression; e.g. *Igf2*) or vice versa (maternal expression; e.g. *Igf2R;*[4]). Differential expression may be more common than complete silencing of one allele and expression of the other. At the molecular level, imprinting is caused by differential DNA methylation and histone modifications or non-coding RNAs that, in mammals, is established early in embryogenesis [5]. At the phenotypic level, genomic imprinting is manifest in differences between reciprocal heterozygotes (which are genetically equivalent in a 2-allele system) as the only difference between these is the parent-of-origin of the two parental alleles [6].

Only about 100 imprinted genes have been formally described in mice (geneimprint.org, March 2012), yet significantly more imprinted loci have been described in quantitative genetic studies (e.g. [7-9]), several of which show complex interaction effects dependent on environmental factors [1,10]. It should be noted, however, the discovery of imprinting effects in studies using allele substitution estimation (of the paternally vs maternally derived allele) in F2 crosses (e.g. [7]) has been criticized for being biased toward the detection of spurious imprinting effects [11]. On the other hand, using DNA sequence characteristics and a machine learning approach, Luedi *et al.*[12] predicted that 600 of over 23,000 annotated genes are imprinted in the mouse. These quantitative genetic studies provide vital information on the location and effects of imprinted genes that may then be followed up in knock-out studies for functional description and evaluation. Perhaps even more important is that these studies illustrate that most imprinted gene effects do not occur in isolation but may show interactions with other genetic or environmental factors, thus establishing the biological network and pathways in which imprinted genes contribute to phenotypic variation. This can be achieved with knock-out studies only within the limited context of one or two loci.

The recognition that imprinting may be more common and important than previously thought has led to an expansion of studies in this field employing quantitative genetic approaches in addition to molecular studies. While early studies have focused on imprinted gene effects that occur during early development [13], more recent work has demonstrated that imprinting effects are not limited to early development [7,9,14] nor are they limited to tissues involved in maternal / fetal interaction. We now know that imprinting can affect body composition traits, growth, behaviour [8,15] as well as cognitive abilities in mice and humans [16]. In addition, studies have now started to estimate how much phenotypic variation is explained by variation at imprinted loci, an indication of how important imprinting may be as a source of variation [17]. For example, Wolf *et al.*[9] showed that pleiotropic imprinting effects can explain up to almost 6% of phenotypic variation alone.

Traditionally, research on imprinting has distinguished only two patterns of expression, namely paternal and maternal expression, and many data bases of imprinted genes follow this convention (e.g. geneimprint.org; http://igc.otago.ac.nz). However, more complex patterns have been discovered [9] with one of the notable examples being the *callipyge* phenotype described in sheep where one of the two heterozygotes shows muscular hypertrophy while the other three genotypes have normal appearance and do not differ from each other [18,19]. Although some pioneering work on imprinting was done in livestock (e.g. [7,20]), the importance of this key epigenetic mechanism for traits of commercial value is only beginning to be recognized in research efforts. However, few studies have been able to clearly separate maternal genetic effects from imprinting effects [21], which can cause the same phenotypic patterns [22], and have assessed how important imprinting is in explaining phenotypic variation compared to genetic sources of variation such as dominance and additive effects.

In this study, we have used a quantitative genetic approach to identify imprinted loci that modulate key obesity and muscle traits (body weight, body lean mass, body fat mass, lactate value, muscle glycogen content and muscle glycolytic potential) in mice derived from the Berlin Muscle Mouse Inbred (BMMI) strains. We first scanned the entire genome for QTL that show epigenetic (causing parent-of-origin phenotypic effects) as well as genetic effects (additive and dominance). Next, we determined whether parent-of-origin-specific effects are due to genomic imprinting or maternal genetic effects as only the former are true epigenetic effects. Finally, the proportion of phenotypic variation accounted for by additive, dominance and genomic imprinting effects was calculated. Our analysis enables us to investigate loci with pleiotropic effects and, at the same time, to investigate whether epigenetic or genetic effects are more important in explaining phenotypic variance in traits of interest.

## Results

**Figure 1:**
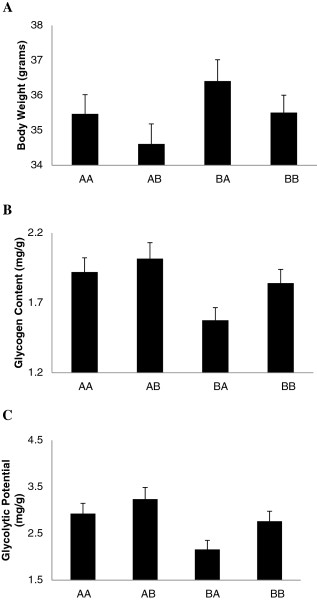
**Imprinting patterns for A) body weight on *Mc12.1,*B) glycolytic content and C) glycolytic potential on the pleiotropic QTL *Mc19.1.*** The pattern of imprinting effect is bipolar expression for all the traits. We have used uncorrected trait means (i.e. before adjustment for direction of cross and sex) to illustrate the magnitude of effects in this figure.

We discovered genomic imprinting effects located on chromosome 19 with a strong effect on glycolytic potential and content. The imprinting pattern was bipolar where the two heterozygotes are different but the two homozygotes are not, consistently for all affected traits (Figure 1). The bipolar pattern is also reflected in the absence of an additive genetic effect, which is required for either paternal or maternal expression [8]. This is somewhat surprising as one might have expected a parental expression pattern (either paternal or maternal expression) since these patterns are commonly assumed to be more prevalent. An additional imprinted locus was found for body weight on chromosome 12. Figure 1 shows two different patterns of bipolar imprinting expression. While body weight shows a pattern with a higher value for the maternal heterozygote (*B/A*), glycolytic potential and glycogen content have higher values for the paternal heterozygote (*A/B*).

A closer look at the genomic structure of the imprinted regions shows that the iQTL at 27.3 Mb on chromosome 12 comprises an informative haplotype block of 8.2 Mb (corresponding to 4.3 cM, [23]) between 26.6 and 34.8 Mb where the alleles differ between BMMI806 and BMMI816. This region is well represented by linkage disequilibrium in the F_3_ population with 0.4 and 3.9 cM on each side of the marker at the peak iQTL position. The next informative regions are 12.7 and 14.2 Mb proximal and distal of the peak marker, respectively, and are only in weak linkage disequilibrium with the peak iQTL marker. With regard to the iQTL on chromosome 19, the marker at the peak iQTL position at 28.9 Mb is located in an informative genomic region of 1.8 Mb (1.7 cM, [23]) between 27.5 and 29.3 Mb. Additional informative genomic regions are located between 24.2 and 24.5 Mb, and 35.2 and 35.5 Mb which are 4.5 Mb (4.2 cM) and 6.3 Mb (5.9 cM) distant from the peak iQTL marker, respectively. However, the marker at 35.2 Mb did not show imprinting effects. Since the informative marker intervals on chromosomes 12 and 19 are small, we have high confidence in the peak locations and genotyping of additional markers would not improve the iQTL detection, neither in respect to position nor significance. Frequencies of all four genotype classes at the peak marker loci are balanced (range: 0.20 to 0.31, Additional file 1: Table S1) so that test statistics are unlikely to be biased.

We detected a further seven QTL on different chromosomes affecting variation at all seven traits (Table 1). Most loci showed pleiotropic effects (e.g. *Mc1.1, Mc2.1, Mc19.1*), however, four loci affected a single trait only (*Mc6.1, Mc12.1, Mc12.2* and *Mc17.1*). Although we identified several loci that affected overall body weight and the correlated traits fat and lean mass (e.g. *Mc1.1*), two loci affected overall body weight only (*Mc12.1* and *Mc17.*1) but not fat mass and lean mass (traits commonly associated with body weight). Six of the nine loci showed clear additive effects, followed by two imprinted QTL and one dominance locus. This pattern is evinced in the number of genetic effects for all traits: additive effects show up 16 times, imprinting effects nine times and dominance effects only five times (Table 1).

**Table 1:** Identified loci with effect scores

**QTL**	**Trait**	**Pos Mb**	**Pos cM**	**C.I cM**	**mLOD**	***a*** **LOD**	***d*** **LOD**	***i*** **LOD**
***Mc1.1***	Body weight	34.6	13.3	13.33 – 44.4	**4.91**	**4.89**	0.88	0.45
	Lean mass			13.33 – 44.4	**3.6**	**3.53**	1.03	0.24
	Glycolytic potential			13.33	0.92	0.56	**1.68**	0.09
	Fat mass			13.33 – 40.2	**2.28**	**3.21**	0.81	0.13
	M. quadriceps			13.33	**1.56**	**2.02**	0.31	0.39
	M. longissimus			13.33 – 26.7	1.13	**1.87**	0.16	0.09
	Muscle mass			13.33	**1.44**	**2.17**	0.21	0.02
***Mc2.1***	M. longissimus	82.0	49.1	49.1 – 54.1	**3.08**	**3.55**	1.23	1.23
	Lean mass			49.1 – 54.1	**2.86**	**3.53**	**1.47**	0.96
	M. quadriceps			49.1 – 54.1	**4.02**	**4.59**	1.06	1.15
	Body weight			49.1 – 54.1	**1.86**	**2.72**	1.24	0.45
	Muscle mass			49.1 – 54.1	**4.35**	**4.94**	**1.46**	1.26
***Mc6.1***	M. longissimus	12.1	5.3	1.8 – 11.2	**3.30**	**3.39**	0.51	**1.36**
***Mc8.1***	M. longissimus	79.4	36.3	33.1 – 36.3	**2.74**	**3.69**	1.14	0.20
	Muscle mass			33.1 – 36.3	**2.94**	**3.97**	1.12	0.09
***Mc12.1***	Body weight	27.3	9.7	9.7 - 28.7	**2.24**	0.21	0.34	**3.30**
***Mc12.2***	Fat mass	91.4	43.9	51.4 – 61.2	**2.17**	**3.30**	0.11	0.01
***Mc15.1***	Muscle mass	10.3	58.7	58.7	**2.57**	0.19	**3.32**	0.13
	M. quadriceps			50.4 - 58.7	**3.29**	0.41	**4.08**	0.01
***Mc17.1***	Body weight	77.6	48.1	58.1 – 60.9	**2.87**	**3.63**	0.07	0.88
***Mc19.1***	Glycolytic potential	28.9	23.5	23.5	**3.91**	0.49	1.30	**3.33**
	Body weight			23.53 – 29.8	**1.77**	1.23	0.09	**1.93**
	Glycogen content			23.53	**2.60**	0.43	0.50	**2.94**
	Lean mass			23.53	**2.21**	1.5	0.01	**2.20**
	M. longissimus			23.53 – 29.8	**1.91**	0.39	0.06	**2.78**
	Muscle mass			23.53 – 29.8	**2.07**	0.40	0.05	**2.82**
	M. quadriceps			23.53 – 38.8	**1.69**	0.39	0.54	**1.87**

M. quadriceps is the summed up mass of the two M. quadriceps, M. longissimus is the mass of the two M. Longissimus, muscle mass is the sum of the two M. longissimus and the two M. quadriceps, fat mass is the body composition’s fat mass and lean mass is the body composition’s lean mass, glycogen content is measured from M. longissimus. Pos Mb is the physical position of the marker in Mb and Pos cM is the position of marker in centiMorgans. LOD scores in bold are significant. Confidence intervals (C.I.) are given in cM using a one LOD drop following Lander & Botstein [24].

Next, we calculated the proportion of phenotypic variation explained by individual loci and their effects (Table 2). Because our QTL model is orthogonal we can add up the proportion explained by individual effects [17]. Table 2 also summarizes the pleiotropic QTL effects observed at most loci by giving the number of loci and their individual effects. Interestingly, variation for glycolytic potential and content are modulated only by genomic imprinting effects. It can be seen that our detected QTL explain up to 40% of phenotypic variation (fat mass). For the majority of traits, additive effects explain most of the variation. However, for body weight and muscle mass, dominance and imprinting effects contribute a significant proportion to the overall variation explained. The imprinted loci have relatively strong QTL effects, individually accounting for 4% to 4.7% of variation.

**Table 2:** Proportion of phenotypic variance explained by all QTL for each trait

**Trait**	**Loci**	***a *** **effects**	***d *** **effects**	***i *** **effects**	**R**^***2***^ ***a***	**R**^***2***^ ***d***	**R**^***2***^ ***i***	**Total R**^***2***^
Body weight	8	7	0	1	26.35	0	2.71	29.06
Muscle mass	7	6	1	0	19.08	4.03	0	23.11
Fat mass	5	4	0	0	39.36	0	0	39.36
Lean mass	7	7	0	0	15.28	0	0	15.28
Glycolytic potential	1	0	0	1	0	0	4.66	4.66
M. quadriceps	5	4	1	0	6.15	1.25	0	7.40
M. longissimus	5	5	0	0	14.60	0	0	14.60
Glycogen content	1	0	0	1	0	0	4.05	4.05

Column two specifies the total number of QTL detected for each trait. Columns 3, 4 and 5 give the QTL with their respective additive, dominance and imprinting effects., *R*^*2*^*a, R*^*2*^*d*, *R*^*2*^*i* and *Total R*^*2*^ denote to the phenotypic variance explained by the, additive effect, dominance effect, imprinting effect and main QTL effect, respectively.

## Discussion

The key result of this study is that genomic imprinting effects but not genetic variation affects glycolytic potential and glycogen content in the BMM mouse lines selected for divergent muscle traits. We discovered a further seven loci with additive and dominance effects that modulate variation in muscle and obesity traits. Overall, variation at our nine loci accounted for up to 40% of phenotypic variation, a considerable amount. There was a clear distinction between the three loci that exert strong pleiotropic effects (*Mc1.1, Mc2.1* and *Mc19.1*) and those that affect only one trait (*Mc6.1, Mc12.1, Mc12.2* and *Mc17.1*).

The results of this study clearly further strengthen the evidence for the role of genomic imprinting at later life stages (e.g. [17,25]): the imprinting effects found here were manifest at 10 weeks of age, well into adulthood for these mice. Moreover, the patterns at the two imprinted loci were not the traditional paternal or maternal expression pattern. Another deviation from previously held views is that imprinted genes are predicted to mainly affect resource transfer between mother and utero (although Haig’s conflict model upon which this view rests has always been more general [26]). However, here we can demonstrate that genomic imprinting affects physiological traits, in line with recent research showing imprinting effects on body composition and other complex traits [2,8].

The confidence regions of our imprinted QTL do not encompass any known imprinted genes (cf. geneimprint.com; March 2012). However, RNA-seqence analysis of brain tissues by Gregg and colleagues [27] revealed a much greater number of loci that show parent-of-origin specific differences in gene expression. Bearing in mind that these data are from different tissues compared to our phenotypes, we do find that our two imprinted loci (*Mc12.1* and *Mc19.1*) are within regions identified by Gregg and colleagues in the hypothalamus and the medical prefrontal cortex (Additional file 2: Table S2). However, the expression patterns for our loci are clearly bipolar whereas Gregg and colleagues only distinguish parental expression patterns. For our tissues, we thus regard our iQTL as novel imprinted loci, which warrant further investigation both in model systems and at the molecular level. However, *Mc1.1* showing additive effects for multiple muscle traits is within the confidence region of previously found additive QTL for body weight, lean mass and fat mass [28], and also in line with the QTL on chromosome 1 for growth found by Brockmann *et al.*[29]. The finding of bipolar imprinting effects on glycogen content and glycogen potential together with the absence of other genetic effects is intriguing, showing that imprinting can play an important role in modulating physiological traits of importance in livestock breeding. Glycogen content and glycolytic potential are determinant factors in meat quality [30], affecting the ultimate pH of meat, which, in turn, affects other quality traits such as water holding capacity, incidence of spoilage [31,32] and meat quality [33]. A mutation in the PRKAG3 gene in Hampshire pigs, for example, causes high glycogen content in skeletal muscle, which has beneficial effects on meat content but detrimental effects on processing yield [34].

Results of our analysis of the proportion of phenotypic variance explained by additive, dominance and imprinting effects contrasts with results of weight traits in a different population generated from mouse lines divergent for body weight at day 60 [17]. In this study, most traits were affected by additive, dominance and imprinting effects with additive effects explaining most of the variation, followed by dominance and imprinting effects. By contrast, the genetic architecture of muscle and fat traits described in the present study is different, although clearly a large part of body weight. Here, few dominance effects (5) were detected, followed by imprinting effects (9), and 16 additive effects across all traits. Moreover, genomic imprinting was the only significant source of variation at glycogen content and glycolytic potential, explaining ~4.5% of variation. This result warrants further investigation, in particular, whether selection for muscle traits may have resulted in selection at imprinted loci. Given that the imprinting pattern is bipolar, i.e. there is no additive effect (difference between homozygotes), it will be intriguing to explore how selection may have resulted in such an imprinting pattern or whether alternative explanations need to be invoked. This point seems particularly relevant to livestock production in which special breeding schemes are often used to benefit from heterosis effects and this paper contributes to encircle relevant regions for commercially important traits.

## Conclusion

Our results show that variation in key physiological traits such as glycogen content is modified by parent-of-origin-dependent gene expression or genomic imprinting but not by genetic variation. These results further demonstrate that imprinting effects are not limited to early developmental phenotypes nor that imprinting occurs predominantly as paternal or maternal expression.

## Methods

### Animals

For this study, we used mice of the Berlin Muscle Mouse (BMM) population, which has been selected for high body weight and muscle mass, primarily in order to investigate the selective mechanisms in livestock breeding [35]. The selection history of BMM has been described previously [28]. We used the BMMI806 and BMMI816 lines, which are hyper-muscular but do not carry the Mstn^Cmptdl1Abc^ mutation. Two pairs of the Berlin Muscle Mouse inbred lines BMMI806 and BMMI816 were crossed reciprocally to generate the F_1_ generation [28]. 94 F_2_ animals were produced that were then randomly mated [36] to produce 345 F_3_ animals. For the analysis of imprinting effects, we used all 94 F_2_ animals and 331 F_3_ animals out of 345 for our QTL analysis because of genotyping errors in some individuals.

### Husbandry

All experimental protocols were approved by the German Animal Welfare Authorities (approval no. G0405/08). The animals were maintained under standard conditions (22 ± 2°C temperature; 12:12 hours light:dark cycle). Two to four animals of the same sex were put in cages with *ad libitum* access to food and water. The animals were fed a standard diet (Altromin standard breeding diet no. 1314 TPF, Lage, Germany) until they were 70 days old. This diet was composed of 27.0% crude protein, 5.0% crude fat, 4.5% crude fibre, 6.5% crude ash, 50.5% nitrogen free extract (starch and sugar), vitamins, trace elements and minerals (2988 kcal/kg metabolizable energy; thereof 27.0% energy from proteins, 13.0% from fat and 60.0% from carbohydrates).

### Phenotypic measures

The mice, at 71 days of age, were anaesthetized by isoflurane and sacrificed after two hours of fasting. The Musculus longissimus (ML) and Musculus quadriceps (MQ) were dissected and weighed, and muscle mass (MM) was recorded as summed muscle weight of left and right M. longissimus and left and right M. quadriceps. The right muscles were promptly frozen in liquid nitrogen and then stored at −80°C. Carcasses were kept at 6 °C and pH values were measured within the M. biceps femoris at 1 and 24 hours post mortem (ebro PHT 810, Ingolstadt, Germany). The glycolytic potential was measured from glycogen content and lactate content [37]. We adjusted the phenotypes for sex and direction-of-cross (Parental Grand Mother) reducing variance not attributed to QTL. Values for total fat and total lean mass were measured by quantitative magnetic resonance (QMR) analysis, using the EchoMRI whole body composition analyser (Echo Medical Systems, Houston, Texas, USA [38,39]). After the two-hour fasting, before dissection, blood glucose levels were measured. We measured the muscle glycogen content colorimetrically in the right M. longissimus (GOD/PAP method ’Glucose liquicolor’ by Human, Wiesbaden, Germany) following Barham and Trinder [40]

### Genotyping

The two parental lines BMM806 and BMMI816 were generated from the same founder population that had been selected for high muscularity over many generations. Since we genotyped both lines with the Mouse Diversity SNP Array [41], comprising 623,124 single-nucleotide polymorphisms (SNPs), we could identify haplotypes that were identical or differed between the two lines. Using the high dense SNP information, we selected 164 informative markers on all chromosomes, except Y. Marker distances in genomic regions that differed between the two parental lines were below 10 Mb, which was about 5 cM (Additional file 3: Figure S1) [23]. A higher marker density would not lead to a higher mapping resolution in this pedigree [42]. Intervals larger than 10 Mb did not contain informative markers and thus could not add information to the linkage analysis.

All animals of the F_2_ and F_3_ generations were genotyped at KBiosciences (Hoddesdon, U.K.). Genotypes were checked for errors and genetic distances with RQTL (http://cran.r-project.org/web/packages/RQTL/index.html). Genotype frequencies at every marker locus are given in Additional file 1: Table S1. Although imprinted genes on the X chromosome have been reported, the imprinting analysis for our model is unresolved at present. The conversion of the physical map into a genetic map was performed using “Mouse Map Converter” software from the Jackson Laboratory.

### QTL analysis

The data analysis for this paper was conducted using SAS 9.1 (SAS Institute, Cary, NC) and SPSS (SPSS Inc., Chicago IL) and PedPhase (version 2.0). We reconstructed haplotypes using Pedphase [43] to produce a set of unordered haplotypes for the F_2_ generation and a set of ordered (by allelic parent-of-origin) haploytpes for the F_3_. We distinguish four ordered genotypes denoted *AA, AB, BA, BB* (paternal / maternal allele) with the *A* allele originating from BMMI806 and the *B* allele from BMMI816. In a first step we assigned the four ordered genotypes at the marker loci additive (*a*), dominance (*d*), and parent-of-origin (*i*) genotypic index scores following Wolf *et al.*[9].

(1) $$\left[\bar{\begin{array}{l} AA\\ \bar{\begin{array}{l} AB\\ \bar{\begin{array}{l} BA\\ \bar{BB} \end{array}} \end{array}} \end{array}}\right]=\left[\begin{array}{cccc} 1 & 1 & 0 & 0\\ 1 & 0 & 1 & 1\\ 1 & 0 & 1 & -1\\ 1 & -1 & 0 & 0 \end{array}\right]\left[\begin{array}{l} r\\ a\\ d\\ i \end{array}\right]$$

This can be solved to yield estimates of the genotypic values

(2) $$\left[\begin{array}{l} r\\ a\\ d\\ i \end{array}\right]=\left[\begin{array}{l} \frac{\bar{AA}}{2}+\frac{\bar{BB}}{2}\\ \frac{\bar{AA}}{2}-\frac{\bar{BB}}{2}\\ \frac{\bar{AB}}{2}+\frac{\bar{BA}}{2}-\frac{\bar{AA}}{2}-\frac{\bar{BB}}{2}\\ \frac{\bar{AB}}{2}-\frac{\bar{BA}}{2} \end{array}\right]$$

*AA*, *AB*, *BA* and *BB* are genotypic values and *r* is the reference point of the model, i.e. the midpoint between homozygotes, *a* gives the additive genotypic value, i.e. half of the difference between two homozygotes, *d* the dominance genotypic value, i.e. the difference between the heterozygote mean and the midpoint of the homozygotes and finally *i* denotes the imprinting value, i.e. half the difference between heterozygotes [1].

To detect QTL we performed a genome scan using a linear mixed model fitted by restricted maximum likelihood (REML) with locus *a*, locus *d* and locus *i* as fixed class variables [9] and family as a random effect variable to control for the background influences of other loci and shared environmental effects.

Significant genome-wide thresholds for each trait were calculated by 1000 permutations [44]. In addition to these genome-wide thresholds, we chose point-wise LOD scores higher than 1.3 (p ≤ 0.05). The latter threshold was applied to detect pleiotropic effects when a locus was significant at the genome-wide level for other traits [8]. QTL are identified when either the overall LOD (mLOD) or the *a, d, i* LOD scores exceeded the genome-wide threshold for a given trait.

Our prior work demonstrated that parent-of-origin dependent effects on offspring phenotypes may be caused by either maternal genetic effects or genomic imprinting [22]. Differences in maternal genotype can cause differences between phenotypes of heterozygous offspring and thus cause the same parent-of-origin effect patterns as those caused by genomic imprinting effects. We therefore tested all loci with a significant parent-of-origin effect to determine whether the effect is due to a maternal genetic effect or genomic imprinting. This was achieved by using a mixed model to test whether the parent-of-origin-dependent effect differed significantly between individuals reared by homozygous versus heterozygous mothers [1].

Finally, the proportion of phenotypic variance explained by a locus was calculated by dividing the genotypic variance (Vg) by the phenotypic variance (Vp) given that REML does not compute sums of squares and the corresponding *R*^*2*^*,* following Hager *et al.*[17]. For additive effects, the explained phenotypic variance by each QTL effect is (1/2 *a*^2^/Vp) × 100; for dominance effects this is given by (1/4 *d*^*2*^/Vp) × 100, and for imprinting effects by (1/2 i2/Vp) × 100.

We denote QTL as Mc, for mouse chromosome, followed by the number identifying the chromosome and the number of a particular locus on a chromosome.

## Competing interests

The authors declare that they have no competing interests.

## Authors’ contributions

ARV, RH and SK conducted the analysis. SK collected pheno- and genotypes. RH, SK and GAB conceived the analysis. All authors contributed to writing the manuscript. All authors read and approved the final manuscript.

## Supplementary Material

Additional file 1 **Table S1.** Number of individuals for each of the four genotypes at each of the markers. Frequencies of the four genotypes at each of the markers. Click here for file

Additional file 2 **Table S2.** Comparison of iQTL with regions showing parent-origin specific gene expression in the hypothalamus (POA) and medical prefrontal cortex (mFPC), given by sex and expression pattern from [27]. Click here for file

Additional file 3 **Figure S1.** Map of reference single nucleotide polymorphisms used in this study.Positions are given in Mb. Bars indicate identified QTL with genome-wide significance. Click here for file
