# Supplementary figures and images for: Genomic imprinting and genetic effects on muscle traits in mice
Source: BMC Genomics. 2012 Aug 20;13:408. doi: 10.1186/1471-2164-13-408 (PMC3475036; doi:10.1186/1471-2164-13-408)

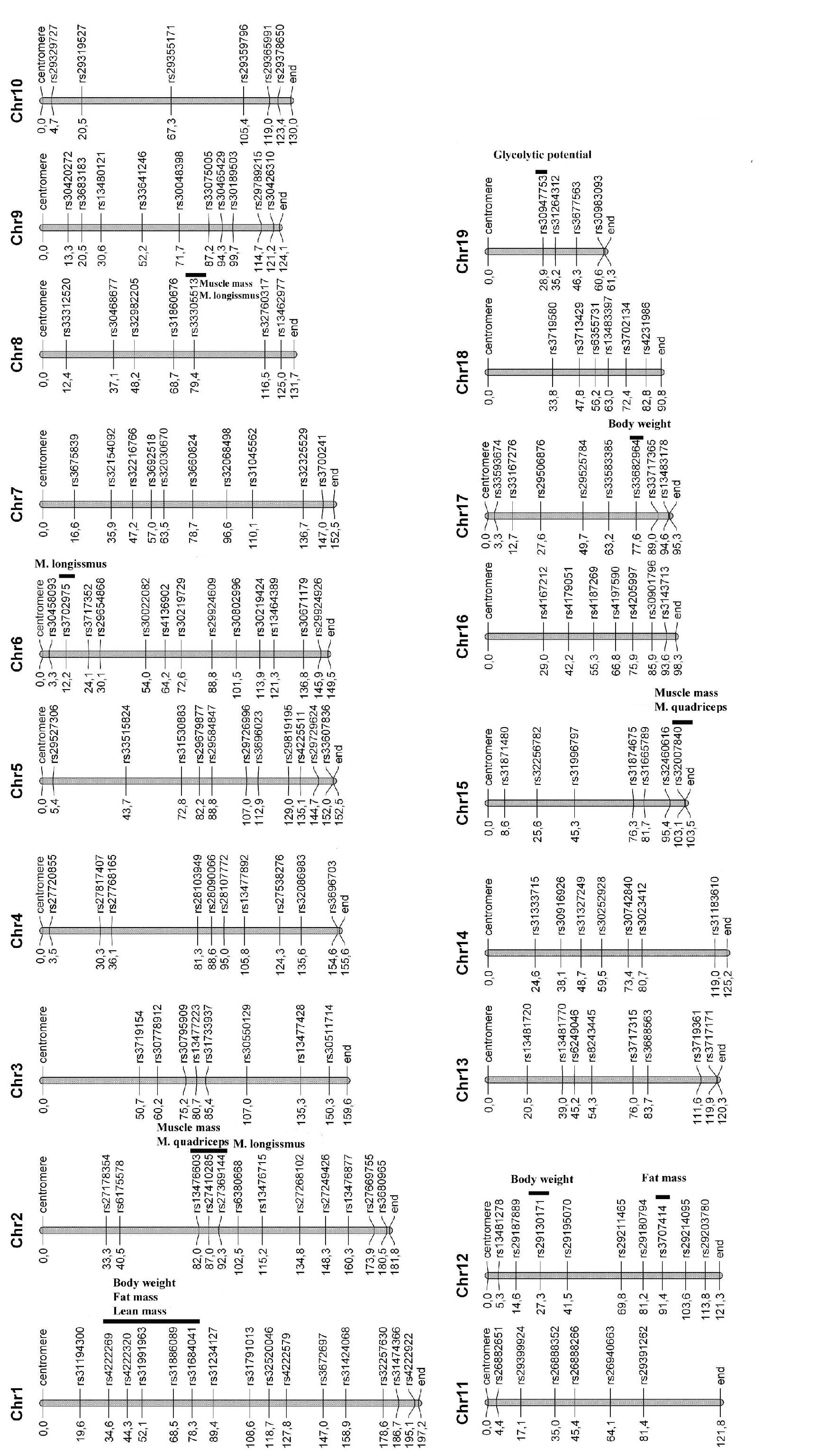
Supplemental Figure 1

Supplement: Additional file 3 — Figure S1. Map of reference single nucleotide polymorphisms used in this study.Positions are given in Mb. Bars indicate identified QTL with genome-wide significance. [file 1471-2164-13-408-S3.doc]
